# Withaferin A protects against palmitic acid-induced endothelial insulin resistance and dysfunction through suppression of oxidative stress and inflammation

**DOI:** 10.1038/srep27236

**Published:** 2016-06-02

**Authors:** Kalaivani Batumalaie, Muhammad Arif Amin, Dharmani Devi Murugan, Munavvar Zubaid Abdul Sattar, Nor Azizan Abdullah

**Affiliations:** 1 Department of Pharmacology, Faculty of Medicine, University of Malaya, 50603 Kuala Lumpur, Malaysia; 2 School of Pharmaceutical Sciences, 11800 Universiti Sains Malaysia, Pulau Pinang, Malaysia

## Abstract

Activation of inflammatory pathways via reactive oxygen species (ROS) by free fatty acids (FFA) in obesity gives rise to insulin resistance and endothelial dysfunction. Withaferin A (WA), possesses both antioxidant and anti-inflammatory properties and therefore would be a good strategy to suppress palmitic acid (PA)-induced oxidative stress and inflammation and hence, insulin resistance and dysfunction in the endothelium. Effect of WA on PA-induced insulin resistance in human umbilical vein endothelial cells (HUVECs) was determined by evaluating insulin signaling mechanisms whilst effect of this drug on PA-induced endothelial dysfunction was determined in acetylcholine-mediated relaxation in isolated rat aortic preparations. WA significantly inhibited ROS production and inflammation induced by PA. Furthermore, WA significantly decreased TNF-α and IL-6 production in endothelial cells by specifically suppressing IKKβ/NF-κβ phosphorylation. WA inhibited inflammation-stimulated IRS-1 serine phosphorylation and improved the impaired insulin PI3-K signaling, and restored the decreased nitric oxide (NO) production triggered by PA. WA also decreased endothelin-1 and plasminogen activator inhibitor type-1 levels, and restored the impaired endothelium-mediated vasodilation in isolated aortic preparations. These findings suggest that WA inhibited both ROS production and inflammation to restore impaired insulin resistance in cultured endothelial cells and improve endothelial dysfunction in rat aortic rings.

Obesity has reached epidemic proportions in the U.S. and it is rising worldwide. Obesity is closely correlated with a state of low-grade inflammation which is characterised by increased levels of proinflammatory cytokines in tissues and blood^1^. Inflammatory reaction is a main feature of reactive oxygen species (ROS) production and insulin resistance in the endothelial cells. Inflammation, ROS and insulin resistance are linked with dyslipidemia, type 2 diabetes, atherogenesis, hypertension, and disorders of blood fibrinolysis and coagulation, all of which are independent risk factors for the progression of atherosclerotic vascular disease, which include strokes, peripheral arterial disease and heart attacks^2^.

Disorders in lipid metabolism play vital part in the progression of endothelial dysfunction in obesity, insulin resistance, and diabetes. An abnormality in patients with all of these disorders is an increase of plasma concentration of free fatty acids (FFA)^2^. Saturated FFA such as palmitic acid induces endothelial inflammation by increasing inhibitor of κB kinase complex β (IKKβ)-nuclear factor (NF)-κB signaling. This comes about via a mechanism that involves production of NADPH oxidase-generated superoxide and toll-like receptors (TLRs), especially TLR4 signaling pathway^3^. Among the consequences of TLR4 induced phosphorylation of NF-κB is the production of proinflammatory cytokines. Activation of IKKβ also impairs vascular insulin signaling and reduces nitric oxide (NO) production, which in turn contributes to accelerating endothelial dysfunction^4^.

Endothelial dysfunction characterized by impairment of endothelium-dependent vasodilatation can result from an increase in ROS and inflammation which will then lead to a loss of insulin-stimulated nitric oxide (NO) secretion. Increased plasminogen activator inhibitor type-1 (PAI-1) and endothelin-1 (ET-1) secretion also contribute to endothelial dysfunction resulting in more expression of adhesion molecules and progression of pro-thrombotic state^3^,^5^. Reduced bioavailability of NO, in part due to increased oxidative stress and inflammation, appears to play a crucial role in the endothelial dysfunction associated with obesity. Indeed, low-grade chronic vascular inflammation is an important event in the pathogenesis of endothelial dysfunction and cardiovascular disease (CVD)^6^. Elevated concentrations of interleukin-6 (IL-6), tumor necrosis factor (TNF-α) and other inflammatory cytokines in plasma have been shown to be linked to endothelial dysfunction in obese patients. Previous study shows that NF-κB activation alters the insulin receptor substrate-1 (IRS-1) phosphorylation thus providing the link between inflammation and insulin resistance^7^.

*Withania somnifera* possesses anti-inflammatory, antioxidant, anti-stress, anti-carcinogenic, anti-aging, cardio-protective, hypothyroid and immunomodulatory activities. *Withania somnifera’s* pharmacological activities have been attributed to its active component, Withaferin A (WA) which is a steroidal lactone^8^,^9^. It has been proposed that the pharmacological actions of WA may be mediated in part through inhibition of NF-κB activation and NF-κB regulated gene product^8^,^9^,^10^. Kaileh *et al.*^10^ identified the steroidal lactone, WA, as the most potent inhibitor of NF-κB based on the hyperphosphorylation of IKKβ which then prevents IκB degradation. Grover *et al.*^11^ suggested that WA inhibits NF-κB by preventing the formation of NF-κB essential modulator IKKβ complex. Furthermore, WA has been shown to be an effective anti-inflammatory compound in cystic fibrosis with potential to treat other chronic inflammatory diseases such as inflammatory bowel disease, rheumatoid arthritis and asthma^12^.

Since oxidative stress and inflammation are purported to play key roles in the development of endothelial insulin resistance and dysfunction in diseases where FFAs are elevated, this study tested the beneficial effect of WA on these pathological conditions associated with PA-induced oxidative stress and inflammation.

## Results

### Effect of Withaferin A on HUVEC’s viability

**Figure 1:**
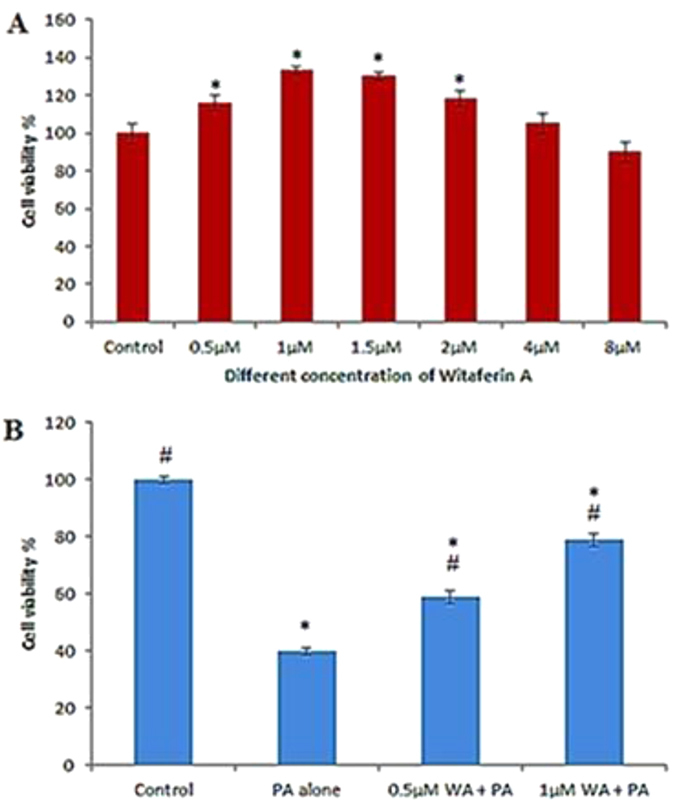
Effect of WA on viability of HUVECs. (**A**) Effect of various concentrations of WA on viability of HUVECs after 24 h. (**B**) Cytoprotective effect of WA on PA (100 μM) induced stress in HUVECs after 24 h pre-treatment. The results are expressed as mean ± SD of three independent experiments. *p < 0.05 vs control, ^#^p < 0.05 vs PA alone.

In order to investigate the effect of WA alone on HUVEC, the cells were treated with different concentrations of WA (0.5, 1, 1.5, 2, 4, 8 μM) and assessed for the number of viable cells after 24 h as the viability declined. It appears that WA increased the cell viability in a concentration-dependent manner, with maximum viability at concentration of 1 μM after which a decline with a significant reduction was noted (Fig. 1A). However, this result should be interpreted with carefulness as it is known that sometimes cell culture imposes a state of oxidative stress on cells that can lead to senescence, cell death, or adaptation^13^,^14^. Withaferin A is a known antioxidant, thus when added to cultured cells, the drug is correcting the oxidative stress effect on cells rather than improving viability. Nonetheless, doses up to 1 μM were selected for further experiments.

### Withaferin A ameliorated HUVEC’s viability under palmitic-induced stress

HUVECs were pretreated with the WA at concentrations of 0.5 and 1 μM for 24 h, following which they were then cultured with 100 μM PA to determine the viability of the cells. Pretreatment with WA concentration-dependently demonstrated higher cell viability (p < 0.05) compared to the cells that were cultured with 100 μM PA alone (Fig. 1B).

### Withaferin A suppressed ROS production in HUVECs

**Figure 2:**
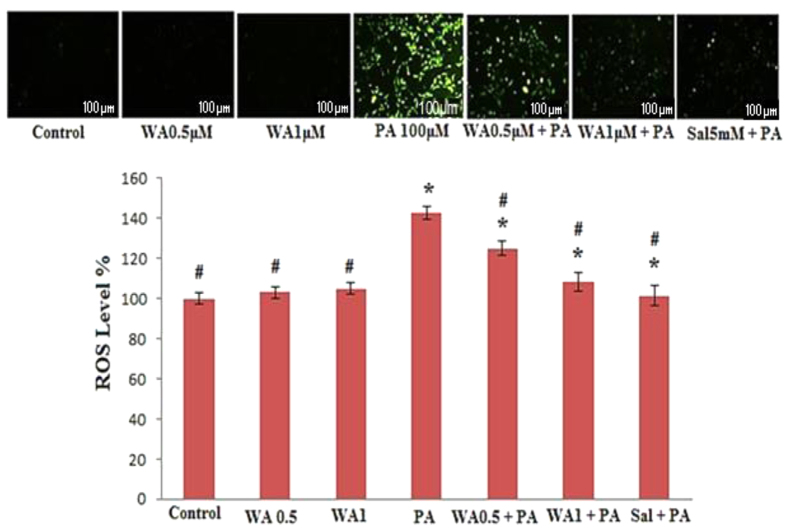
ROS scavenging effect of the WA. Representative images (**A**) and summarized results (**B**) of cells exposed to WA (0.5 and 1 μM) or salicylate (5 mM) for 24 h, then stimulated without or with PA (100 μM) for 24 h. The results are expressed as mean ± SD of three independent experiments. *p < 0.05 vs control, ^#^p < 0.05 vs PA alone.

Treatment with WA alone did not produce ROS (Fig. 2). Cells exposed to PA (100 μM) for 24 h demonstrated an increased fluorescence intensity indicating enhanced ROS production. However, pretreatment with WA (0.5 and 1 μM) negated PA-stimulated ROS production. The suppression action of WA at concentration of 1 μM was comparable to that of 5 mM salicylate (Fig. 2).

### Withaferin A inhibited PA- induced IKKβ and NF-kB activation and decreased TNF-α and IL-6 production in HUVECs

**Figure 3:**
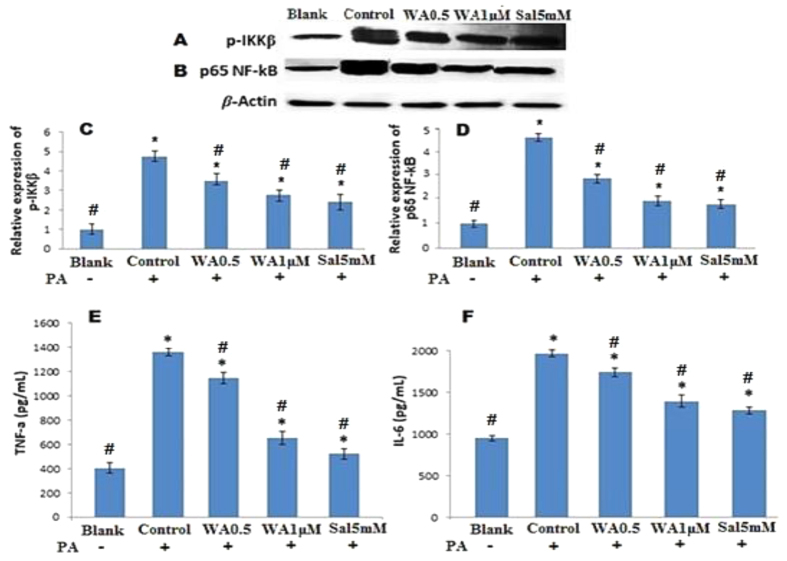
WA inhibited IKKβ/NF-κB signaling in PA treated HUVECs. Representative blots and relative expression of phosphorylated IKKβ (**A,C**) and p65 NF-kβ (**B,D**) in cells exposed to WA (0.5 and 1 μM) or salicylate (5 mM) for 24 h, then stimulated without or with PA (100 μM) for 24 h. TNF-α (**E**) and IL-6 (**F**) levels from medium of HUVECs pretreated with WA, salicylate for 24 h, and then exposed to PA (100 μM). Salicylate was used as positive control. The results are expressed as the mean ± SD of three independent experiments. *p < 0.05 vs blank, ^#^p < 0.05 vs control.

IKKβ/NF-κB signaling regulates inflammation. PA stimulation caused the phosphorylation of NF-κB (Fig. 3A,C) and IKKβ (Fig. 3B,D). WA pretreatment inhibited the activated IKKβ phosphorylation and completely inhibited NF-κB phosphorylation by inhibiting p65 activation. Salicylate also demonstrated a similar inhibitory action on IKKβ and NF-κB phosphorylation elicited by PA stimulation. IKKβ/NF-κB controls the expression of a broad range of pro-inflammatory cytokines, including IL-6 and TNF-α. As shown in Fig. 3E,F, when HUVECs were stimulated with PA the production of IL-6 and TNF-α were greatly increased. Treatment with WA effectively inhibited PA stimulated IL-6 and TNF-α production. Salicylate also strongly decreased TNF-α and IL-6 secretion stimulated by PA (Fig. 3E,F).

### Withaferin A modulated insulin receptor substrate-1(IRS-1) serine/tyrosine phosphorylation in the presence of PA

**Figure 4:**
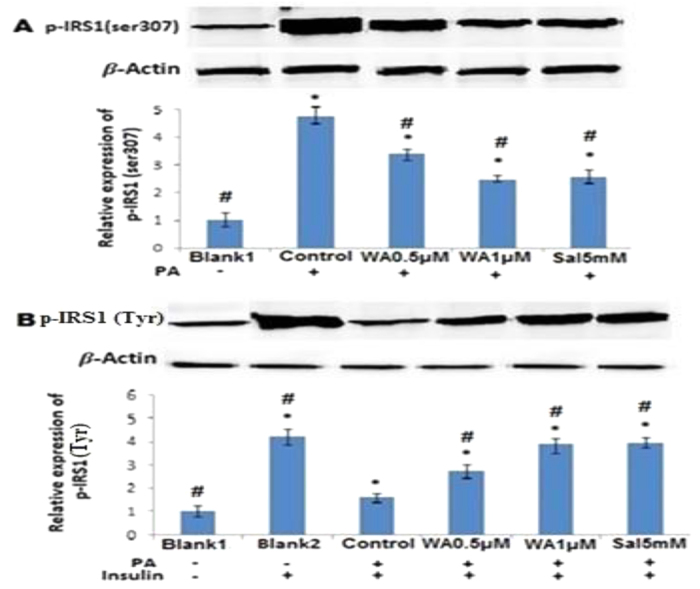
Effects of WA on IRS-1 serine/tyrosine phosphorylation in PA treated HUVECs. (**A**) Representative blot and relative expression of Serine (S307) phosphorylation of IRS-1 in cells incubated with WA (0.5 and 1 μM) and, salicylate (5 mM) for 24 h, and then stimulated without or with PA (100 μM) for 24 h. (**B**) Representative blot and relative expression of tyrosine phosphorylation of IRS-1 in cells stimulated with PA, followed by treatment with insulin (100 nM). Salicylates were taken as positive controls. Results are expressed as mean ± SD of three independent experiments. *p < 0.05 vs blank 1, ^#^p < 0.05 vs control.

PA stimulation induced phosphorylation of IRS-1(serine 307) (Fig. 4A) but blocked IRS-1 tyrosine (Fig. 4B) phosphorylation in the presence of insulin. Exposure of endothelial cells to WA at concentrations of 0.5 and 1 μM increased IRS-1 tyrosine phosphorylation mediated by insulin (Fig. 4B) and reduced serine phosphorylation of IRS-1 (Fig. 4A). Salicylate also demonstrated same effects on IRS-1 tyrosine and serine phosphorylation as WA.

### Withaferin A modulated on insulin mediated Akt and eNOS phosphorylation and NO production in the presence of PA

**Figure 5:**
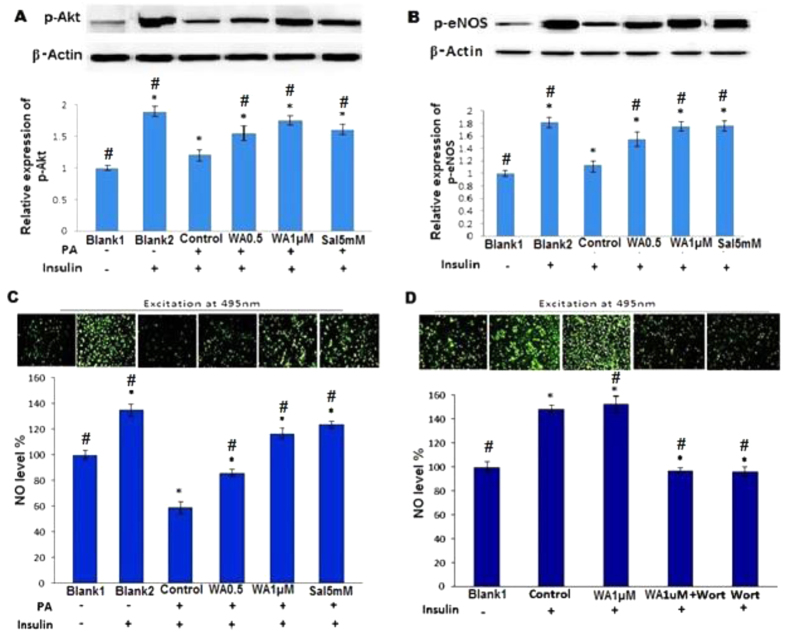
WA restored the decreased insulin mediated Akt and eNOS phosphorylation and NO production in PA-treated endothelial cells. (**A,B**) Representative blots and relative expression of p-Akt (**A**) and p-eNOS (**B**) in HUVECs pretreated with WA (0.5 and 1 μM) or salicylate (5 mM) and then exposed with 100 μM PA, followed by addition of insulin (100 nM). (**C**) Representative images and summarized results of fluorescence imaging of intracellular NO production by fluorescence microscopy of HUVECs pretreated with WA or salicylate, then incubated with 100 μM PA, followed by insulin (100 nM) stimulation. (**D**) Representative images and summarized results of fluorescence imaging of intracellular NO production by fluorescence microscopy of HUVECs were pretreated with 1μM WA, then incubated with 100 nM wortmannin (wort), followed by insulin (100 nM). The data are expressed as the mean ± SD of three independent experiments. ^∗^p < 0.05 vs Blank1, ^#^p < 0.05 vs Control.

**Figure 6:**
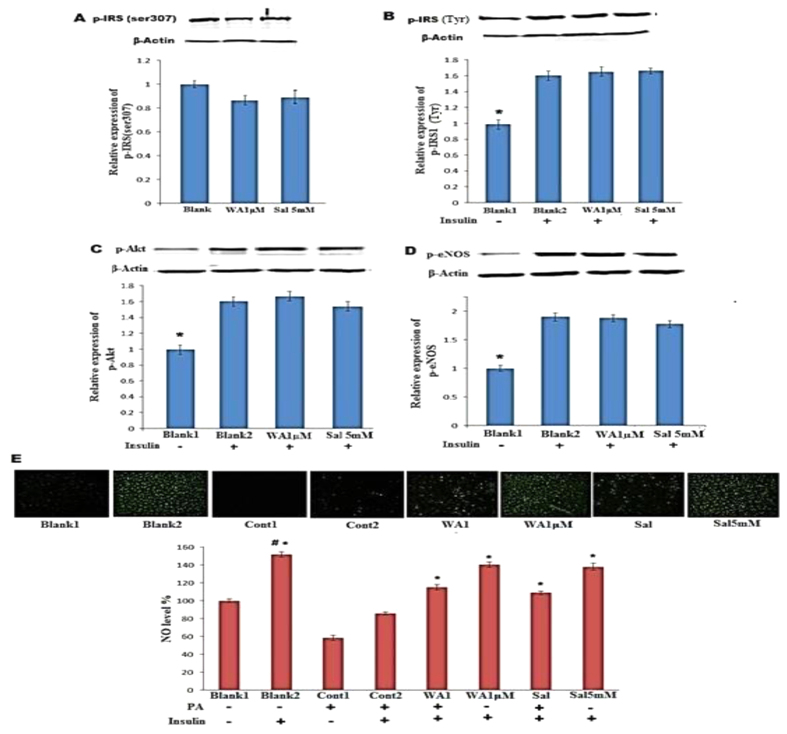
Effect of Withaferin A on insulin signaling with and without PA stimulation. (**A**) Representative blots and relative expression of phosphorylated IRS at Ser307 in HUVECs pretreated with 1 μM WA or salicylate (Sal, 5 mM). Representative blots and relative expression of phosphorylated IRS-1 at Tyr (**B**), phosphorylated Akt (**C**) and phosphorylated eNOS (**D**) in HUVECs pretreated with 1 μM WA or 5 mM salicylate for 30 min followed by addition of insulin. The data presented in (**A**–**D**) was expressed as the mean ± SD of three independent experiments. *p < 0.05 vs Blank 2. (**E**) Representative images and summarized data of intracellular NO production of HUVECs pretreated with 1 μM WA or 5 mM salicylate, then incubated with or without 100 μM PA before incubated with 100 nM insulin. The data was expressed as the mean ± SD of three independent experiments. ^#^p < 0.05 vs Blank1, *p < 0.05 vs control 2.

PA exposure inhibited insulin mediated IRS-1 tyrosine phosphorylation, which will lead to the deterioration of downstream insulin signaling signified by decreased Akt and eNOS phosphorylation in the presence of insulin. WA pretreatment effectively mitigated the inhibitory effect of PA on insulin-mediated Akt/eNOS phosphorylation (Fig. 5A,B). As illustrated in Fig. 5C,D, insulin mediated NO production from HUVECs was blocked by PA treatment. WA effectively restored insulin stimulated NO production and blocked PA impact. Insulin stimulated NO production was inhibited by Wortmannin (specific inhibitor of PI3K), but pretreatment of WA (Fig. 5D) does not affect this change. WA did not alter IRS-1 function (Fig. 6A,B), phosphorylation of Akt (Fig. 6C) and eNOS (Fig. 6D) as well as NO production (Fig. 6E) without PA stimulation. PA stimulation decreased NO production induced by insulin and this inhibition was reversed by WA (0.5 and 1 μM), and salicylate (Fig. 6E). This suggests that facilitation of insulin PI3K signaling by WA is an indirect effect due to inhibition of the inflammatory pathway.

### Withaferin A inhibited ET-1 and PAI-1 production in the endothelial cells

**Figure 7:**
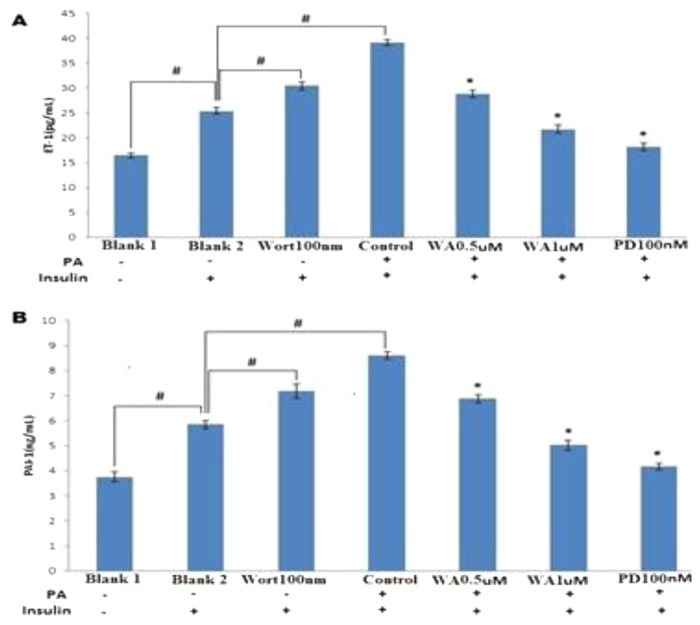
WA reduced ET-1 and PAI-1 levels in PA-stimulated endothelial cells. (**A**) ET-1 and (**B**) PAI-1 levels measured with ELISA from cell medium pretreated with WA (0.5 and 1 μM), selective MAP/ERK kinase inhibitor, PD98059 (PD, 100 nM) or selective PI3 kinase inhibitor, wortmannin (Wort, 100 nM) and incubated with PA (100 μM) followed by stimulation with or without insulin (100 nM). Salicylate was taken as a positive control. The results are expressed as the mean ± SD of three independent experiments. ^#^p < 0.05 vs Blank2 (Bla2), *p < 0.05 vs control.

Insulin supports the production of ET-1 and PAI-1 through MAPK/ERK pathways in endothelial cells. ET-1 and PAI-1 levels were increased following addition of insulin and this action was enhanced by PI3 kinase inhibitor, wortmannin. When endothelial cells were exposed to PA, ET-1 and PAI-1 levels were further enhanced in response to insulin. Interestingly, WA effectively inhibited the overproduction of ET-1 and PAI-1. PD98059, a specific inhibitor of MAPK/ERK kinase, also decreased ET-1 and PAI-1 levels which shows the involvement of ERK activation (Fig. 7A,B, respectively).

### Withaferin A restored impaired acetylcholine-mediated relaxation of aorta induced by PA

**Figure 8:**
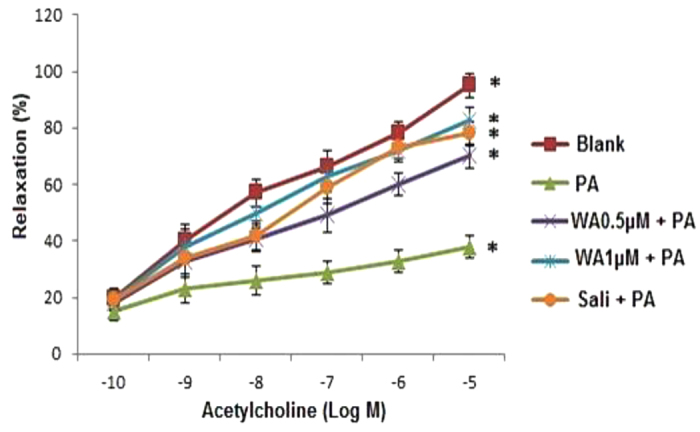
WA restored PA induced loss of endothelium-dependent vasorelaxation in the rat aorta. Concentration response curve to ACh-induced relaxation of aortic rings following pretreatment with WA (0.5 and 1 μM) or salicylate (5 mM) for 30 min, followed by PA (100 μM) for 30 min. Salicylate was used as a positive control. The results are expressed as the mean ± SD of six independent experiments. *p < 0.05 vs PA.

ACh induced vasodilation at concentrations ranging from 0.1 nM to 10 μM. PA (100 μM) decreased ACh-induced vasodilation, with maximal relaxation at 10 μM ACh reduced from 85% to 55%. WA (0.5 and 1 μM) reversed PA-induced impairment of ACh-mediated vasodilation in a concentration-dependent manner with maximal relaxations at 10 μM ACh restored to 85% and 90%, with WA 0.5 μM and 1 μM, respectively (Fig. 8). Salicylate (5 mM) also showed the same effect as WA and the maximal relaxation was restored to 78%. In aortic tissues, insulin (1 nM to 10 μM) did not induce relaxation (data not shown).

## Discussion

Elevated FFAs is implicated in obesity-associated endothelial insulin resistance and dysfunction. The mechanism underlying this link is cellular inflammation at the level of IKKβ/NF-κB signaling and ROS production is required for FFA to induce this signaling pathway. Among the consequences of PA-induced inflammation in endothelial cells is impaired insulin signaling and reduced nitric oxide production which is purported to precede the development of atherosclerosis. Given that vascular inflammation is a key mechanism linking cardiovascular disease to obesity and other metabolic disorder, heightens the need for therapeutic intervention that not only targets FFA-induced inflammation but also the associated oxidative stress.

WA is an antioxidant with potent inhibitory action on NF-κB and thus would be a good strategy to target multiple pathways that contribute to endothelial insulin resistance and dysfunction associated with raised levels of FFA. The present study was undertaken to investigate whether WA could suppress PA-induced production of ROS and inflammation and consequently diminish endothelial insulin resistance and dysfunction. We report that in cultured HUVECs, WA concentration-dependently inhibited PA induced oxidative stress. Furthermore, WA blocked PA-induced IKKβ/NF-κB activation and upregulated IRS-1 tyrosine phosphorylation, which facilitated insulin signaling transduction along the IRS-1/PI3K/Akt/eNOS pathways. Conversely ET-1 and PAI-1 levels were suppressed. Finally in rat aorta, WA alleviated endothelial dysfunction elicited by PA.

It is well-established that FFAs can cause inflammation and insulin resistance in liver, skeletal muscle and endothelial cells^15^. One possible mechanism whereby elevated FFAs induce cellular inflammation is via ROS production such as superoxide that can be generated by mitochondrial uncoupling^16^,^17^,^18^. Other study suggested that PA-induced endothelial inflammation is dependent on nicotinamide adenine dinucleotide phosphate (NADPH) oxidase-generated superoxide via a mechanism that involves Toll-like receptor (TLR) activation^3^. In this regard, our study corroborated that PA stimulation increased ROS production in endothelial cells and besides, pretreatment with WA significantly reduced the PA-induced ROS production. As PA-induced ROS production is mediated by NADPH oxidase, it cannot be ruled out that WA may inhibit this enzyme. However, this requires further investigation. Furthermore, pretreatment with WA, significantly improved the reduced cell viability observed in the presence of PA. Therefore, the present finding also demonstrated a strong correlation between the suppression of ROS production by WA with the decreased cytotoxicity induced by PA in HUVECs.

In obesity, an increased efflux of FFAs from adipocytes leads to activation of pro-inflammatory signaling pathways via ROS. Increased oxidative stress is associated with stimulation of IKKβ and activation of transcription factor NF-κB which in turn mediates an inflammatory response by increasing expression of several pro-inflammatory cytokines including TNF-α, IL1-β, IL6 and MCP-1^19^,^20^,^21^. In the present study, PA induced IKKβ and NF-κB p65 phosphorylation resulted in higher level of TNF-α and IL-6 generation in endothelial cells. Some of these pro-inflammatory cytokines can signal through their cognate receptors to activate IKKβ and might then sustain endothelial inflammation^22^. WA has been reported to suppress the production of inflammatory mediators and protect against cytokine induced cell damage while improving cell survival in cancer study^23^. Moreover, WA has been described to inhibit inflammation in macrophages by blocking NF-κB pathway^24^. Similarly, the present study showed that treatment of cultured endothelial cells with WA concentration-dependently abolished PA induced IKKβ/NF-κB phosphorylation, and suppressed production of IL-6 and TNF-α. These effects were comparable to salicylate, an IKKβ inhibitor, thus indicating WA anti-inflammatory action may be in part due to suppression of IKKβ/NF-κB activation and inflammatory cytokines, TNF-α and IL-6 production. Based on the present finding, it is conceivable that WA suppressed inflammation indirectly by suppressing ROS production and also independently of its antioxidant effect. Moreover, WA was reported to potently inhibit NF-κB activation by preventing TNF-α induced activation of IKKβ via a thioalkylation-sensitive redox mechanism^10^.

Insulin resistance defined by defective action of insulin on target organs is a common feature of obesity; and elevated level of FFAs is the key element for the development of both inflammation and insulin resistance not only in metabolic tissues but also in the endothelium^2^,^25^,^26^. Besides predicting type 2 diabetes mellitus, insulin resistance is an important risk factor for the development of cardiovascular diseases and this is probably related to the effect of insulin resistance on endothelial function^25^,^26^. Impairment of PI3-K signaling pathway is a characteristic feature of insulin resistance whilst other insulin-dependent branches such as Ras/MAPK pathways are unaffected^27^,^28^. Specific impairment of PI3-K signaling pathway reduces insulin stimulation of glucose uptake in muscle and fat tissue whereas in endothelial cells, the capacity of insulin to stimulate nitric oxide production is reduced^28^,^29^. When insulin binds to its receptors on endothelial cells, tyrosine phosphorylation of insulin receptor substrate-1 (IRS-1) occurs which subsequently activates PI3-K. The activated PI3-K then stimulates downstream Akt and eNOS, which initiates NO production^30^. Inflammatory cytokines such as TNF-α, IL-6 and IKKβ activation can increase serine phosphorylation of IRS-1, with subsequent reduction in insulin-dependent tyrosine phosphorylation of IRS-1 thereby impairing PI3K/Akt/eNOS pathway of insulin signaling^19^,^20^. The present study demonstrated that PA blocked the phosphorylation of IRS-tyrosine by inducing IRS-1 serine phosphorylation in HUVECs in response to insulin. Additionally, our study demonstrated that PA reduced insulin-mediated phosphorylation of Akt and eNOS in cultured endothelial cells. WA pretreatment prevented the effects of PA by inhibiting IRS-1 serine phosphorylation and increasing insulin stimulated IRS-1 tyrosine phosphorylation thus allowing activation of insulin PI3-K/Akt/eNOS signaling pathway. The improvement of insulin Akt/eNOS phosphorylation by WA may well be a consequence of its positive modulation of IRS-1 phosphorylation against PA induced oxidative and inflammatory insult since inhibition of insulin stimulated NO production by wortmannin, a PI3-K inhibitor, is not reversed by WA.

This study also demonstrated that insulin-stimulated ET-1 and PAI-1 levels were increased in endothelial cells following impairment of PI3-K signaling in the presence of PA. Likewise, insulin-stimulated ET-1 and PAI-1 levels were elevated in endothelial cells following inhibition of PI3-K by wortmannin. Taken together, these results proved that when PI3-K was inhibited, MAPK/ERK-dependent actions of insulin were enhanced with subsequent increase production of ET-1 and PAI-1. WA pretreatment perhaps in part through its suppression of ROS production and inflammation, restored insulin-mediated NO production in a PI3K-dependent manner and concomitantly inhibited ET-1 and PAI-1 production. It appears that WA restored the balance between PI3-K- and MAPK- dependent functions of insulin in endothelial cells when challenged with PA. These findings confirmed an earlier study that generated similar results^31^.

Obesity and insulin resistance are associated with endothelial dysfunction which is specifically in part due to abnormalities in the endothelium derived nitric oxide system^32^. In essence, endothelial dysfunction results from an imbalance in the release of vasodilators such as NO and vasoconstrictors such as ET-1^33^. Alteration in this balance predisposes the endothelium towards an atherogenic environment^34^. Under insulin resistant state, it is proposed that abnormal vascular insulin signaling induces endothelial dysfunction, characterized by attenuated nitric oxide-mediated vasodilatation^33^,^35^. Since the usual parameter assessed when testing endothelial function is endothelium-dependent relaxation, we thus set out to study whether insulin could induce vascular relaxation in rat aortic rings and how this relaxation would be compromised in PA-induced insulin resistance to eventually explore the beneficial effects of WA. However, the present study unlike previous study^31^, failed to demonstrate that insulin could induce vasodilatation in rat aortic rings. The reason for this discrepancy is not certain, but other studies have also failed to demonstrate insulin mediated vasodilatation^36^,^37^. These studies reported that it was unclear whether the link between insulin and NO production was localized to a specific intracellular signal transduction pathway or, conversely, all endothelium-mediated responses can be influenced by insulin^36^. It is well-known that in addition to NO-dependent action, insulin has other actions that influence haemodynamic homeostasis. Insulin also secretes ET-1 from endothelium which is a potent vasoconstrictor^38^. Indeed, Arcaro *et al.*^39^, showed evidence that hyperinsulinaemia could give rise to severe endothelial dysfunction in humans especially in large conduit arteries, which are prone to develop atherosclerosis. Thus it appears that insulin has opposing actions which helps to maintain the haemodynamic balance between the vasodilator (NO) and the vasoconstrictor (ET-1)^38^, this probably explains the negligible net effect of insulin on vascular reactivity in the present study. Nonetheless, a shift in balance towards the vasoconstrictor action of insulin may be a crucial factor in the vascular pathophysiology of insulin resistance.

Our study further exhibited that in the presence of PA, ACh-mediated vasodilation in rat aortic rings was reduced and WA was found to negate this impairment. Therefore, it is speculated that the restoration of ACh-mediated vasodilation by WA may derive from its suppression of oxidative stress and inflammatory signaling pathways. Although FFA is expected to induce endothelial dysfunction by way of insulin resistance caused by ROS-associated inflammation, oxidative stress and inflammation per se can promote endothelial dysfunction. Evidence suggesting oxidative stress and inflammation alter endothelium functions including modulation of vasomotor tone are plenty. ROS quenches NO with formation of peroxynitrite^40^, which is a cytotoxic oxidant. Consecutively, peroxynitrite can cause nitration of proteins which will affect protein function and therefore endothelial function. Moreover, peroxynitrite leads to degradation of the eNOS cofactor tetrahydrobiopterin (BH4)^41^ leading to “uncoupling” of eNOS. Oxidative stress is also linked to a proinflammatory state of the vascular wall, and inflammatory cytokines are known to play a role in the vascular dysfunction associated with vascular disease. Inflammation decreases NO bioavailability and indeed, C-reactive protein (CRP) has been shown to decrease eNOS activity^42^,^43^. Furthermore TNF-α inhibits endothelium-dependent NO-mediated dilation by ceramide-induced activation of JNK^44^,^45^,^46^ and TNF-α treatment of rat mesenteric arteries results in inhibition of NO-dependent relaxation^47^.

Overall, our study suggests that WA is a potent antioxidant and anti-inflammatory substance which can protects the endothelium against PA-induced oxidative stress and inflammation, events that play significant roles in the development of endothelial insulin resistance and dysfunction. Diosgenin, a steroid sapogenin, also possessed similar pharmacological properties to WA, wherein it reversed endothelial dysfunction associated with insulin resistance through an IKKβ/IRS-1-dependent manner^31^. However, this compound, on the contrary to WA, was not reported to have additional antioxidant activity. FFA-induced endothelial insulin resistance and dysfunction are complex disorder. Since both ROS and inflammation seem to play major roles in the development of endothelial insulin resistance and dysfunction, employing a substance such as WA which targets both ROS and inflammation may be a good strategy to correct this disorder and hence prevents the risk of cardiovascular events. The question then arises whether NF-κB can be effectively targeted by WA to inhibit obesity induced inflammation and thus its related cardiovascular diseases since this would necessitate long term treatment and the factor is required for basic immune responses. It seems commonsensical to use NF-κB inhibitors only for short-term therapy in acute situations; however some non-steroidal anti-inflammatory drugs (NSAIDs) or glucocorticoids also block NF-κB and have been used long-term thus suggesting that long-term use of NF-κB inhibitors is a reasonable strategy. Further investigation is necessary to determine whether WA is a specific NF-κB inhibitor and therefore would be devoid of the adverse effects associated with drugs such as NSAIDs and glucocorticoids. Development of such a specific inhibitor of NF-κB could offer significant potential for treatment of obesity related diseases.

## Materials and Methods

### Cell culture

Human umbilical vein endothelial cells (HUVECs) were obtained from Cell Applications, USA and cultured in Dulbecco’s Modified Eagle Medium (DMEM) supplemented with 10% (vol/vol) fetal bovine serum (FBS), 100 U/mL penicillin and 100 U/mL streptomycin and passaged according to the recommended procedures of Cell Applications, USA (CRL-1777). After confluence, the cells were starved for 12 h in serum-free medium containing 0.5% bovine serum albumin (BSA) before drugs or palmitic acid (PA) treatment.

### Cytotoxicity test

The CellTiter 96 Aqueous One Solution Cell Proliferation Assay kit from Promega (Madison, WI) was used for the 3-(4,5-dimethylthiazol-2-yl)-5-(3-carboxymethoxyphenyl)- 2-(4-sulphophenyl)-2H-tetrazolium (MTS) assay following the instruction of the manufacturer. Cells were seeded in a 96-well plate at 1 × 10^5^ cells/well and allowed to adhere for 48 h, and then the cells were pretreated with WA for 24 h and incubated for another 24 h with 100 μM PA. After adding 20 μl of MTS reagent to each well that contained 100 μl medium, the cells were cultured further for another 4 h, and the absorbance was read at 490 nm.

### Measurement of reactive oxygen species (ROS)

The production of intracellular reactive oxygen species (ROS) was measured by using 2′7′-dichlorodihydrofluorescein diacetate (DCFH-DA) reagent^35^. The DCFH-DA reagent enters the cells passively where it is de-acetylated by the esterase to a non-fluorescent DCFH. Inside the cell, DCFH reacts with ROS to form DCF. After changing the culture medium, various concentrations of WA were added. Cells were incubated for 24 h with or without WA, and followed by an additional 24 h incubation in a media containing 100 μM of PA. After washing, the cells were treated with 5 μM of DCFH-DA for 30 min, and the readings were taken at 485-nm excitation and 530-nm emission in a fluorescence plate reader. To calculate the amount of intracellular ROS produced, the mean control treatment was subtracted from the mean treatment group.

### Measurement of intracellular ROS in individual cells, by image analysis

Cells (5 × 10^5^) were seeded on to coverslips in 6-well plates and incubated for 48 h for attachment. On the next day, the medium was replaced with fresh medium containing WA and left for 24 h. Then, the media was replaced with media containing 100 μM concentration of PA. At the end of incubation, the coverslip was removed from the culture plate and stained with 40 μM of DCFH-DA for 30 min. Excess dye was removed by washing with 1 × PBS. The coverslip was mounted on glass slide, and images of the cells were captured using 40× objectives under fluorescence microscope^37^.

### Enzyme-linked immunosorbent assay (ELISA)

The cells (2 × 10^5^ cells/well) were seeded in wells of a 24-well plates and pretreated with WA (0.5 and 1 μM) for 24 h, and then stimulated with PA (100 μM) for further 24 h. The levels of TNF-α and IL-6 were measured in the supernatant by using commercial ELISA Kits (SunRed Biological Technology). For determination of ET-1 and PAI-1 production, HUVECs were treated with WA and then with selective inhibitor of MAP/ERK-kinase, PD98059 and specific inhibitor of phosphoinositide (PI) 3-kinase, wortmannin for 30 min. After that, the cells cultured with PA (100 μM) followed by stimulation with 100 nM of insulin for 30 min and the supernatant was collected to analyses the level of ET-1 and PAI-1 with ELISA Kits (SunRed Biological Technology).

### Detection of NO production

HUVECs were grown in 96 well plates and pretreated with salicylate (positive control) or WA at different concentrations and exposed to 100 μM PA. The cells were then washed out with PBS, exposed to insulin for 5 min and fixed in 2% paraformaldehyde (vol/vol). NO was measured by following incubation at 37 °C for 30 min in the dark with NO-specific fluorescent dye 3-Amino, 4-aminomethyl- 2′,7′-difluoresceindiacetate (DAF-FM DA, Beyotime, Shanghai, China). Fluorescence intensity of the fixed cells were examined using an Inverted Research Microscope with attached charge-coupled device camera (Nikon, ECLIPSE TI-S) using appropriate filters with a peak excitation wavelength of 495 nm and a peak emission wavelength of 515 nm^36^.

### Western blot analysis

After treatment with WA, salicylate, PA or insulin, cells were washed twice with PBS and lysed in mammalian cell lysis buffer (Sigma MCL-1) supplemented with protease and phosphatase inhibitors. Insoluble materials were eliminated by centrifugation (12,000 × g, 10 min, 4 °C). The protein concentration in the supernatant was determined by Bradford assay (Bio-Rad Laboratories). Thirty microgram of protein extracts were loaded on 10% SDS-polyacrylamide gel, and transferred to activated nitrocellulose membrane. The membrane were blocked with tris-buffered saline (TBS) containing 5% nonfat milk, and incubated with primary antibodies against phospho Akt (T308), phospho IRS-1(Ser307), phospho IRS-1(Thr), phospho IKKβ (Y199), phospho-p65 NFkB (Ser536), phospho eNOS (Ser1177) and PY99 (obtained from Santa Cruz Laboratories) overnight at 4 °C. β-actin was used as a loading control. The following day, the membranes were washed in TBS followed by incubation with appropriate horseradish peroxidase–conjugated secondary antibodies for 1 h at room temperature. The membranes were developed using chemiluminescence substrate according to the manufacturer’s instructions (Amersham Life Sciences, Little Chalfont, U.K.). Quantitative analysis of the protein was performed by Gel Documentation System (Biospectrum 410, UVP).

### Preparation of aortic rings and assessment of endothelium-dependent relaxation

Male Sprague–Dawley rats (weighing 200–250 g), were purchased from the Animal Experimental Unit (AEU), University of Malaya. All experimental procedures were performed in accordance to Guide for the Care and Use of Laboratory Animals (Guide) approved by the University of Malaya Animal Care and Ethics Committee and accredited Association for Assessment and Accreditation of Laboratory Animal Care International (AALAC). As reported previously^48^, rats were sacrificed by carbon dioxide (CO_2_) inhalation. The thoracic aorta was immediately removed and placed in 4 °C Krebs-Henseleit (K-H) solution (NaCl 118.3, KCl 4.7, MgSO_4_ 1.2, KH_2_PO_4_ 1.2, CaCl_2_ 2.5, NaHCO_3_ 25, glucose 5.0, Calcium Disodium Edetate (EDTA) 0.026 mM, pH 7.4), and gassed with 95% O_2_–5% CO_2_. The dissected aortas were cleaned of connective tissues and cut into rings (2 mm long). Care was taken to avoid abrading the intimal surface to maintain the integrity of the endothelial layer. For measurement of vascular responses, aortic rings were immersed in an organ bath containing 5 mL of K-H solution maintained at 37 °C, pH 7.4, and continuously aerated with 95% O_2_ and 5% CO_2_. 1.0 g of resting tension was enforced and changes in tension of aortic ring were analyzed with a force-displacement transducer linked to a PowerLab Recording System (AD Instruments, Australia). After 60 min equilibration period, the viability of the rings was tested by addition of 60 mM of KCl repeatedly until reproducible contractions were achieved. The functionality of vascular endothelium was confirmed by the addition of 1 μM phenylephrine into the organ chamber, followed by 10 μM acetylcholine (ACh) before the actual experiments. After confirming the integrity of the endothelium (the relaxation was over 80%), the aortic ring was pre-contracted with phenylephrine (1 μM) and then ACh (0.1 nM to 10 μM) was added cumulatively to elicit an endothelium-dependent relaxation. In another set of experiments, the aortic ring was exposed to WA for 30 min followed by the addition of PA (100 μM) for another 30 min prior to constructing ACh-induced vasodilatation. The relaxation induced by ACh was expressed as a percentage of the phenylephrine-induced contraction.

### Statistics analysis

Results are presented as means ± standard deviation (SD) for number of experiments. Concentration-response curves were fitted to a sigmoidal curve using non-linear regression with the aid of the statistical software GraphPad Prism version 6, (USA). Protein expression was quantified by densitometer (Biospectrum 410, UVP), normalized to β-actin and then compared with control. Data were analyzed with one-way ANOVA followed by Bonferroni post-hoc test using SPSS version 16.0 software. The differences at level *p < 0.05 were considered to be statistically significant.

## Additional Information

**How to cite this article**: Batumalaie, K. *et al.* Withaferin A protects against palmitic acid-induced endothelial insulin resistance and dysfunction through suppression of oxidative stress and inflammation. *Sci. Rep.* **6**, 27236; doi: 10.1038/srep27236 (2016).
